# Psychological eHealth Interventions for Patients With Cardiovascular Diseases: Systematic Review and Meta-Analysis

**DOI:** 10.2196/57368

**Published:** 2025-04-07

**Authors:** Jing Jing Su, Rose Lin, Ladislav Batalik, Arkers Kwan Ching Wong, Sherry L Grace

**Affiliations:** 1 School of Nursing Tung Wah College Hong Kong China (Hong Kong); 2 Translational Research Center for Digital Mental Health Tung Wah College Hong Kong China (Hong Kong); 3 Elaine C. Hubbard Center for Nursing Research on Aging School of Nursing University of Rochester Rochester, NY United States; 4 Department of Physiotherapy and Rehabilitation Faculty of Medicine Masaryk University Brno Czech Republic; 5 Department of Rehabilitation University Hospital Brno Brno Czech Republic; 6 School of Nursing Hong Kong Polytechnic University Hong Kong China (Hong Kong); 7 Faculty of Health York University Toronto, ON Canada; 8 KITE-Toronto Rehabilitation Institute University Health Network University of Toronto Toronto, ON Canada; 9 Peter Munk Cardiac Centre University Health Network University of Toronto Toronto, ON Canada

**Keywords:** cardiovascular diseases, eHealth, digital health, iCBT, mental health, psychological intervention, cognitive behavioral therapy, CBT, depression, heart, cardiology, psychological, anxiety, high-risk, systematic review, meta-analysis, CVD, evidence-based, psychosocial, GRADE approach, Cochrane Risk of Bias Tool, internet-based, psychological therapy, psychotherapy

## Abstract

**Background:**

Psychological distress is recognized as an independent risk factor for cardiovascular diseases (CVDs), contributing to increased morbidity and mortality. While eHealth is increasingly used to deliver psychological interventions, their effectiveness for patients with CVDs remains unclear.

**Objective:**

This meta-analysis aimed to evaluate the effects of eHealth psychological interventions for patients with CVDs.

**Methods:**

Eligible studies were retrieved from 5 databases (Embase, Medline, PubMed, CINAHL, and Cochrane Library), covering the period from database inception to December 2024. Randomized controlled trials (RCTs) investigating the effect of evidence-based psychological eHealth interventions to improve psychosocial well-being and cardiovascular outcomes for people with CVDs were included. The Cochrane Risk of Bias tool (version 2) was used to judge the methodological quality of reviewed studies. RevMan (version 5.3) was used for meta-analysis.

**Results:**

A total of 12 RCTs, comprising 2319 participants from 10 countries, were included in the review. The results demonstrated significant alleviation of depressive symptoms for patients receiving psychological eHealth intervention compared to controls (number of paper included in that particular analysis, n=7; standardized mean difference=–0.30, 95% CI –0.47 to –0.14; *I*^2^=57%; *P*<.001). More specifically, in 6 trials where internet-based cognitive behavioral therapy was delivered, a significant alleviation of depressive symptoms was achieved (standardized mean difference=–0.39, 95% CI –0.56 to –0.21; *I*^2^=53%; *P*<.001). There was no significant change in anxiety or quality of life. Synthesis without meta-analysis regarding stress, adverse events, and cardiovascular events showed inconclusive findings.

**Conclusions:**

Psychological eHealth interventions, particularly internet-based cognitive behavioral therapy, can significantly reduce depressive symptoms among patients with CVDs. A multidisciplinary approach is crucial for comprehensively improving psychological and cardiovascular outcomes. Future studies should explore integrating persuasive design features into eHealth and involving mental health professionals for intervention delivery.

**Trial Registration:**

PROSPERO CRD42023452276; https://www.crd.york.ac.uk/PROSPERO/view/CRD42023452276

## Introduction

The interplay between cardiovascular diseases (CVDs) and psychological distress, including stress, anxiety, and depression, presents a profound challenge in medical care [1]. Notably, approximately 40% of patients with CVDs experience these psychological symptoms [2,3], which not only impair their quality of life but also double their risk of severe cardiovascular events such as myocardial infarction, stroke, hospitalization, and premature death compared to those without such psychological disorders [4,5]. These disorders exacerbate cardiovascular risks through multiple plausible biological mechanisms [6]. Autonomic nervous system dysfunction, triggered by psychological stress, leads to an imbalance favoring the sympathetic nervous system. This chronic activation results in elevated heart rate and blood pressure, reducing heart rate variability and increasing the risk of cardiovascular complications such as hypertension. Additionally, psychological distress activates the hypothalamic-pituitary-adrenal (HPA) axis, increasing cortisol production. Chronic stress can induce cortisol resistance, leading to unregulated inflammation marked by elevated levels of proinflammatory cytokines such as C-reactive protein, interleukin-6, and tumor necrosis factor-α [7]. This persistent inflammation contributes to the exacerbation of atherosclerosis, enhancing the likelihood of arterial plaque instability and rupture, thereby significantly increasing the risk of cardiovascular events [8].

Effectively managing these psychological symptoms is essential for reducing overall cardiovascular risk and enhancing patient outcomes, yet it is complex, primarily due to poor adherence to prescribed treatments [9]. While both pharmacological and nonpharmacological interventions have been shown to improve psychological well-being, the high costs, adverse side effects, and cessation challenges associated with psychoactive medications often hinder patient use [10,11]. Additionally, the complexity of drug regimens and the delayed onset of benefits contribute to poor compliance, particularly in populations already burdened with managing chronic cardiovascular conditions.

Nonpharmacological interventions, serving as a vital form of secondary prevention, have increasingly been used to improve psychological outcomes in individuals with or at risk for CVDs. Techniques such as cognitive behavioral therapy, mindfulness, problem-solving therapy, and stress management do not involve direct costs of medication and are devoid of the side effects associated with drugs, such that they are perceived as more acceptable to many patients [12]. These approaches also address psychological symptoms by teaching patients coping strategies and resilience skills, which are crucial for both immediate and long-term management of stress, anxiety, and depression. Moreover, nonpharmacological treatments can be tailored to individual needs and integrated into patient lifestyles more flexibly than medication regimes, enhancing adherence. They often include group sessions, which can reduce stigma by normalizing psychological challenges among peers. A comprehensive systematic review and meta-analysis of 35 trials highlighted that these interventions not only reduce cardiovascular mortality but also lead to significant improvements in symptoms of depression, anxiety, and stress among patients with coronary heart disease compared to usual care [13].

Despite their proven efficacy, the implementation of these interventions is frequently hindered by limited awareness and resource constraints [5,14]. Therefore, increasing education about these options for both health care providers and patients and ensuring broader accessibility are essential steps toward overcoming these hurdles. The inherent adaptability of nonpharmacological interventions makes them particularly well-suited to meet diverse patient needs, ultimately conserving health care resources and improving long-term health outcomes [15].

eHealth has gained increasing popularity as a means to deliver psychological interventions for CVDs in a scalable and accessible way. eHealth refers to the use of information and communication technology to support health care delivery [16]. Psychological eHealth interventions offer notable advantages: they maintain user anonymity, thereby reducing the fear of stigmatization; eliminate travel time and associated costs; and allow for tailoring interventions to individual preferences, needs, and unique contextual factors [17,18]. Additionally, the interactive capabilities of eHealth platforms are particularly valuable due to the intense, persistent, and fluctuating nature of mental health challenges in patients with CVDs [19,20]. This variability necessitates continuous self-assessment and monitoring to effectively track progress and prevent relapse. eHealth interventions facilitate real-time support from health care professionals, assisting in patients’ active engagement [21], overcoming geographical barriers [22], and promptly identifying nonadherence and subsequently addressing it [23].

There is an emerging body of primary research exploring the effects of psychological eHealth interventions in CVDs [24], such that it is now possible to conduct a first comprehensive systematic review and meta-analysis to synthesize this evidence and inform future care recommendations. This systematic review and meta-analysis, therefore, aims to investigate the impact of eHealth psychological interventions on psychological and cardiovascular outcomes in patients with or at high risk of CVDs.

## Methods

### Design

This review followed the *Cochrane Handbook for Systematic Reviews of Interventions* to formulate the research question, define eligibility criteria, conduct the comprehensive literature searches, assess risk of bias, and synthesize evidence [25]. The PRISMA (Preferred Reporting Items for Systematic Reviews and Meta-Analyses) statement informed the reporting structure of this manuscript [26], ensuring all the key components of a high-quality systematic review were included, including that search strategies and data extraction methods were clearly outlined. The protocol of this review was registered in PROSPERO (CRD42023452276) to enhance transparency, minimize bias, and prevent duplication of effort.

### Eligibility Criteria

are shown in Textbox 1. Inclusion criteria were guided by the populations, interventions, comparisons, outcomes, and study designs (PICOS) framework. Feasibility results were not used as eligibility criteria, as this study aims to investigate the effectiveness of the intervention. However, we extracted this information to inform future research.

**Inclusion and exclusion criteria.**

**Inclusion criteria**

- Population: studies that included individuals with a medical diagnosis of cardiovascular disease (CVD), including arrhythmia, heart failure, valve disease, and cardiomyopathy, as well as studies that included a mixture of patients with CVD and at high CVD risk. High CVD risk was defined as having at least 2 well-established CVD risk factors, including BMI >30 kg/m2 and dyslipidemia in the past month [27]
- Intervention: participation in an evidence-based psychological therapy (eg, cognitive behavioral therapy) delivered through an eHealth platform (eg, websites or apps) that enabled structured therapy delivery
- Comparison: standard care, waitlist control, placebo, or other active controls (eg, health education)
- Outcomes: measures of psychological well-being (eg, depressive symptoms, anxiety, stress), health-related quality of life, and adverse events or cardiovascular events
- Study design: randomized controlled trials were included to ensure rigor [28]

**Exclusion criteria**

- Studies with a quasi-experimental design, conference abstracts, or case studies, to ensure focus on robust experimental methodologies
- Studies with a total sample size of less than 30, due to insufficient power to detect a true intervention effect [29]
- Studies that solely used text messages or phone calls as the intervention method, to prioritize comprehensive eHealth strategies, which are characterized by multimedia and interactive features that significantly enhance user engagement
- Studies evaluating interventions based on psychological principles but primarily aimed at improving adherence to other treatments, such as tobacco cessation, to ensure our review’s focus was on direct psychological outcomes
- Studies without full texts, despite efforts to contact authors, to ensure that our analysis was based on fully accessible data

### Search Strategy

A comprehensive literature search was conducted across 5 electronic databases, covering the period from database inception to December 2024: Embase (from 1910), Medline (from 1946), PubMed (from 1997), CINAHL (from 1981), and the Cochrane Library (from 1996). The selection of these databases aligned with the research question by ensuring broad coverage of medical, psychological, and health-related literature, maximizing the likelihood of identifying relevant studies. The search strategy used a combination of Medical Subject Headings (MeSH) terms and free-text keywords to capture variations in terminology. Boolean operators were applied to refine the search, combining terms such as “cardiovascular disease” AND (“psychological intervention” OR “eHealth”) AND (“depression”). The search strategies for all databases are provided in Multimedia Appendix 1. Additionally, a hand search of bibliographies from relevant review articles was performed to identify any further studies [30].

### Study Selection

Endnote (version X9; Clarivate) was used to identify and remove duplicate records; 2 reviewers (JJS and RL) independently screened studies by evaluating titles and abstracts against the predefined eligibility criteria, focusing on the relevance to the research question and the predefined PICOS criteria. Studies that met the inclusion criteria or had unclear eligibility progressed to a full-text review. Disagreements between the 2 reviewers throughout the review process were resolved through discussion and, when necessary, adjudicated by a third researcher (LB). The third researcher’s role ensured objectivity in the decision-making process and the transparency of study selection.

### Study Quality and Risk of Bias

Methodological quality was assessed independently by 2 researchers (JJS and RL) using the Cochrane Risk of Bias tool (version 2), evaluating each study across 5 domains: randomization, intervention deviations, missing data, outcome measurement, and selective reporting. Bias risk was categorized as “low,” “some concerns,” or “high.” Studies with “some concerns” in 3 or more domains were deemed high risk. Due to the limited number of studies, funnel plots were not generated. Publication bias was evaluated by comparing outcome variables presented in randomized controlled trials with their trial registry and published protocols.

### Data Extraction

Study data were extracted independently by 2 authors (JJS and RL). The authors developed a table for data extraction, which included the following: (1) origin of the articles, including authors, year, and country; (2) sample characteristics, such as gender, sample size, age, setting, and diagnosis; (3) trial design, consisting of a brief description of the intervention and control groups and study duration; (4) data collection time points; and (5) feasibility data regarding recruitment, attrition, and adherence.

### Recruitment, Attrition, and Adherence

The overall rates of recruitment, attrition, and intervention adherence were documented using median, minimum, and maximum values. Predefined cutoff values for the feasibility criteria, specifically a recruitment rate of at least 20% and an attrition of less than 25%, were determined based on clinical relevance as established in a previous study [31].

### Data Analysis and Synthesis

Review Manager (version 5.3; Cochrane) was used for data pooling due to its capacity for combining outcomes from multiple studies with varying sample sizes. Data pooling was conducted when ≥3 studies were reporting the same outcome; otherwise, synthesis without meta-analysis (SWiM) was conducted [32]. The SD, if unavailable, was calculated from the CI. Psychological well-being was considered the primary outcome. Where the same study reported outcomes at multiple end points, those from immediately after the intervention were used to investigate the intervention effect. For continuous outcomes, intervention effects were expressed as standardized mean differences (SMDs) using Cohen *d*, which standardizes differences between groups, enabling comparison across studies with different measurement scales. Cohen *d* values were interpreted as representing small (0.2-0.5), moderate (0.5-0.8), or large (>0.8) effect sizes, which provided a framework to understand the magnitude of an intervention’s impact on psychological outcomes. For dichotomous outcomes, intervention effects were summarized as risk ratios using the Mantel-Haenszel method, which accounts for study weights in pooled analyses and is appropriate for binary data.

Statistical heterogeneity was assessed using the *I*^2^ statistic, which quantifies the proportion of variability in effect estimates due to between-study differences rather than chance. *I*^2^ values were interpreted as follows: 0% to 25% indicated low heterogeneity, 26% to 50% indicated moderate heterogeneity, and >50% indicated substantial heterogeneity. Random-effects models were used when substantial heterogeneity was detected to provide more conservative estimates where heterogeneity was present. Leave-one-out sensitivity analysis was conducted where significant heterogeneity was observed to identify the impact of individual studies on the overall findings by systematically excluding one study at one time to detect outliers that disproportionally contributed to heterogeneity. Due to the limited number of studies, meta-regression and subgroup analysis were not performed [25].

## Results

### Overview

**Figure 1:**
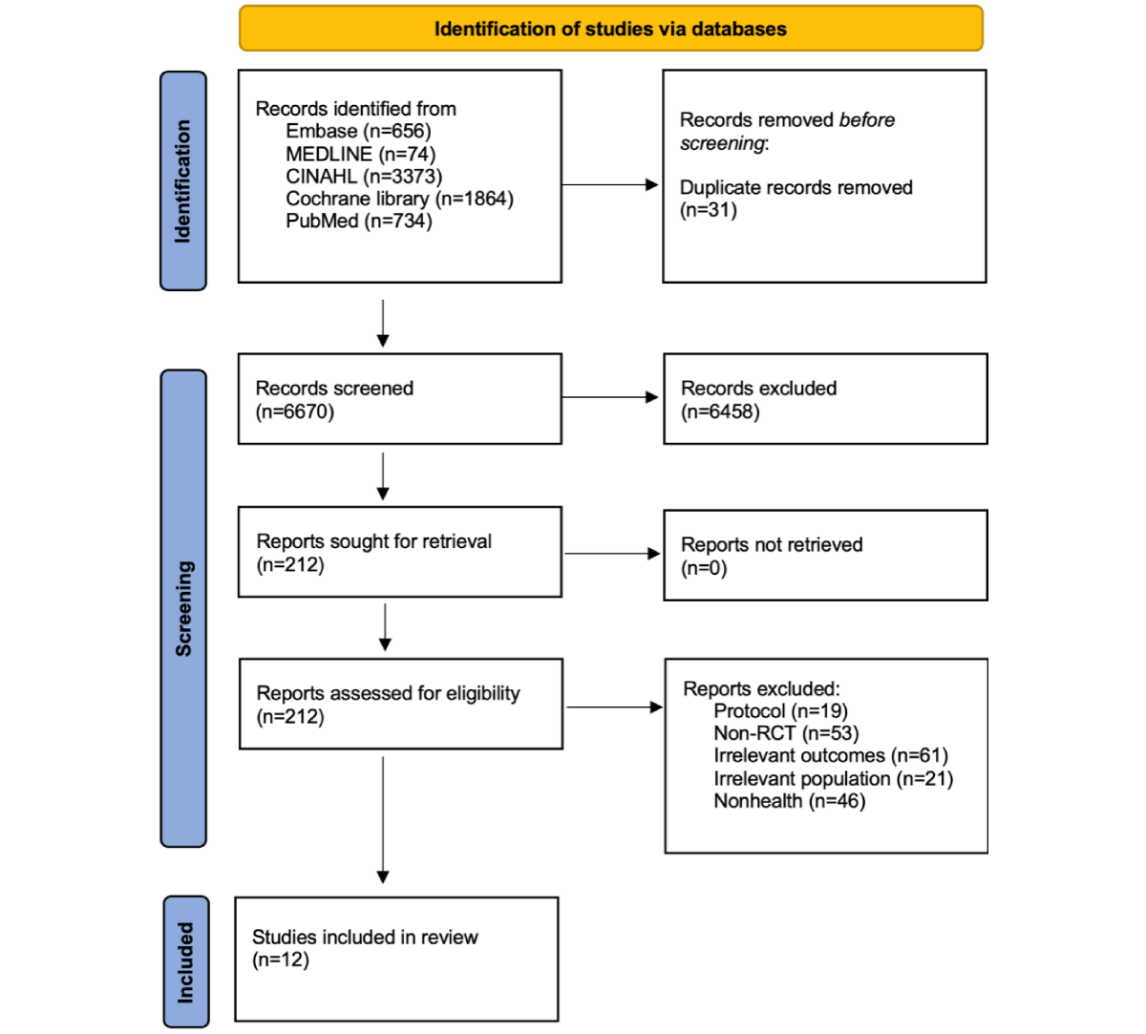
PRISMA (Preferred Reporting Items for Systematic Reviews and Meta-Analyses) flow diagram. RCT: randomized controlled trial.

Figure 1 presents the PRISMA flow diagram illustrating the search results. Initially, 6701 articles were identified across the 5 databases. Following the removal of 31 duplicates, 6670 studies remained for relevance assessment. After screening titles and abstracts, the full texts of 212 studies were examined for eligibility. Ultimately, 12 trials were included in this review.

Multimedia Appendix 1 provides an overview of the included articles. Trials were conducted across 10 countries in Asia, Europe, and North America, including a total of 2319 participants, with study sample sizes ranging from 50 to 562. Participants had a mean age of 58.68 (SD 5.85) years, with a majority being male (62.8%). Participants were diagnosed with heart failure (111/2319, 4.79%) and [24,33] coronary heart disease (628/2319, 27.08%) [34-36], and there was a mixture of patients with CVDs and at high CVD risk (562/2319, 24.2%) [37], controlled arrhythmia (491/2319, 21.17%) [38-40], and general CVDs (527/2319, 22.72%) [31,41,42]. Eight trials focused on individuals with mild to moderate levels of anxiety or depression (1561/2319, 67.31%) [31,33-38,41]. Outcomes were assessed immediately after the interventions. Long-term follow-up data, ranging from 1 to 5 years after the interventions, was only collected in 3 studies [34,38,39].

### Risk of Bias

**Figure 2:**
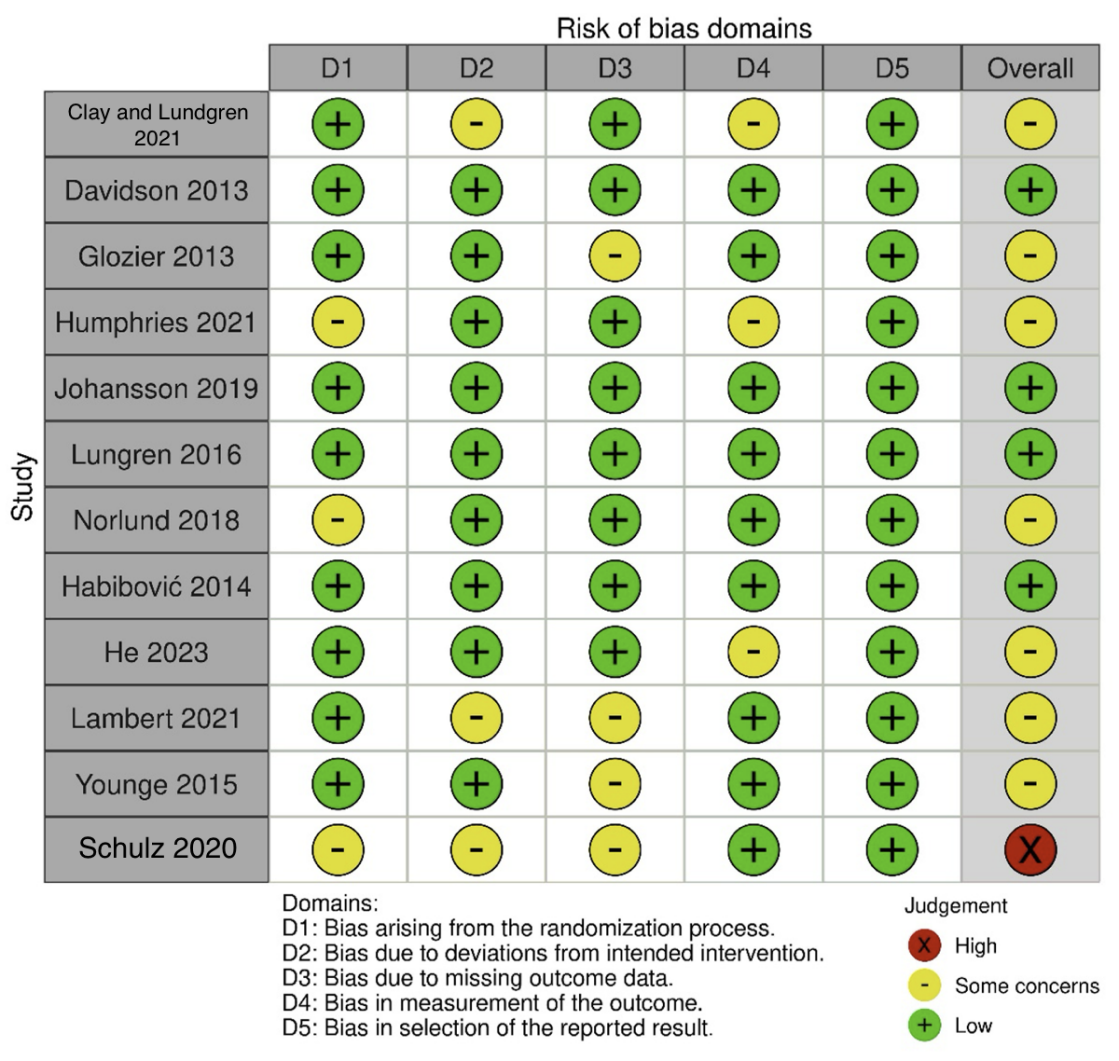
Risk of bias assessment [24,31,33-42].

Figure 2 shows the results of the risk of bias assessment. Most trials were at low risk regarding random sequence generation, deviations from intended interventions, and missing outcome data. “Some concerns” were related to inadequate reporting. Specific issues around detection bias were noted in trials by He et al [40], Humphries et al [34], and Clays et al [24], who explicitly stated that blinding was not implemented due to the behavioral nature of interventions. This lack of blinding raises concerns about potential bias in outcome measurement, as participants and data assessors may have been influenced by knowledge of intervention assignments. Selection bias was consistently rated as low across all studies. In summary, although some studies were categorized as low risk, the presence of high or unclear risks in certain domains necessitates a cautious interpretation of the compiled results. In addition, we recommend adherence to standardized reporting practices using the CONSORT (Consolidated Standards of Reporting Trials) guidelines, which provide a structured framework for transparent reporting of study design, procedures, and outcomes, reducing the potential for bias and improving replicability.

### Intervention characteristics

Four types of psychological interventions were examined across the 12 trials, most commonly internet-based cognitive behavioral therapy (ICBT) [24,33-38,41], followed by mindfulness-based interventions [40,42], problem-solving therapy [39], and stress management [31].

For ICBT interventions, most incorporated core CBT techniques like behavioral activation, worry management, and problem-solving. Specific areas such as psychoeducation [33,35] or the psychosocial aspects of implantable cardioverter defibrillators [38] were the focus of some interventions. Additionally, certain programs integrated additional therapeutic methods, including interpersonal therapy [37] or mindfulness [24]. The interventions used a variety of platforms, from multimedia supports to telemonitoring devices and expert systems for automated guidance. Some featured interactive web-based group settings with discussion forums and secure email systems for queries. For instance, one study provided customization options that allowed participants to tailor their treatment paths after completing compulsory modules [34].

Mindfulness interventions also differed in their approaches. One used a brain-computer interface app with real-time electroencephalography monitoring to facilitate dynamic monitoring over a 35-minute mindfulness exercise, including body scanning and relaxation techniques [40]. The other mindfulness intervention offered a more traditional 12-week online course, encompassing various meditation types, like self-reflection and yoga, supported by biweekly reminders [42]. The 12-week problem-solving therapy, focusing on psychoeducation, used biweekly emails to foster adherence [39]. For stress management, a 2-stage strategy was adopted: participants initially engaged in either a self-guided or lay-coach–supported web program, with nonresponders escalated to a more intensive program that included motivational interviewing [31].

The interventions were delivered by a diverse group of professionals and nonprofessionals. In 4 trials, the intervention was delivered by mental health professionals, including a mental health nurse [33] and psychologists [35,38,39]; in other studies, the intervention was delivered by a multidisciplinary team [24,36], a therapist [34], nurses [40,41], a trained lay coach [31], and, finally, only a self-directed approach in 2 studies [37,42].

### Recruitment, Attrition, and Adherence

Recruitment and attrition rates were considered acceptable [31]. The included trials showed an overall recruitment rate of 42% (ranging from 10% to 85%). Regarding attrition, the median attrition rate was 19% (ranging from 5% to 24%) in intervention groups and 8% (ranging from 3% to 43%) in control groups. In the intervention groups, there were 231 dropouts out of 1213 participants, with 6 (4%) dropouts due to health-related reasons and 225 (96%) due to unspecified non–health-related reasons. In the control groups, there were 108 dropouts out of 1106 participants, with 3 (6%) due to unspecified health-related reasons and 105 (94%) due to non–health-related reasons. It is noteworthy that unspecified non-health reasons were the most common cause of dropouts in both the intervention and control groups (Multimedia Appendix 1).

Adherence to the eHealth interventions varied across the interventions, ranging from 20% to 73%; 2 studies did not report the adherence rate [36,42]. Key factors contributing to low adherence included technical issues (eg, insufficient computer literacy) [24,34], time constraints, and emotional discomfort evoked by the intervention. In addition, one study found that coach calls, originally designed to last 10 to 15 minutes, often exceeded this time frame due to technical challenges [31].

### eHealth Intervention Effectiveness

#### Depressive Symptoms

**Figure 3:**
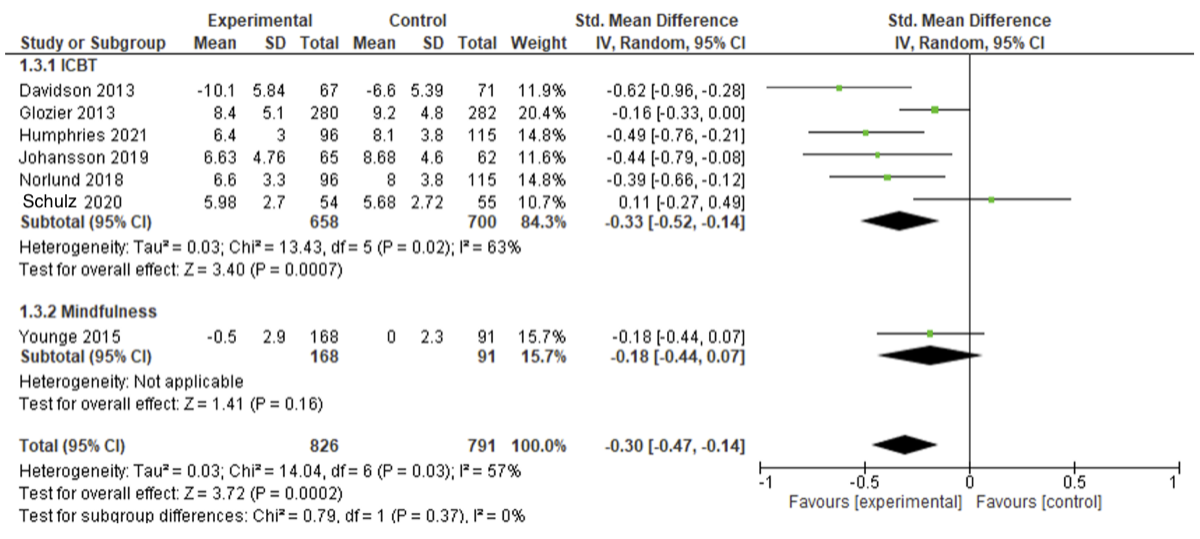
Forest plot showing the efficacy of eHealth psychological interventions for depression.

Figure 3 shows a forest plot for the pooled results on depression. Depression was measured by 11 studies using the Hospital Anxiety and Depression Scale (HADS) [34,35,38,42], Beck Depression Inventory [24,36], Patient Health Questionnaire–9 [33,37,39,41], or the Depression, Anxiety, and Stress Scale (DASS) [31]. Data pooling of 7 trials revealed significant reduction in depression symptoms for participants receiving psychological eHealth intervention compared with control groups receiving usual care or active control (online education [37] and web-based discussion forums [41]; standardized mean difference [SMD]=–0.30, 95% CI –0.47 to –0.14; *I*^2^=57%; *P*<.001). Sensitivity analysis of the 6 studies that provided ICBT revealed significant improvement in depression compared to control groups (SMD=–0.39, 95% CI –0.56 to –0.21; *I*^2^=53%; *P*<.001). Data pooling of 5 trials that compared psychological eHealth interventions with usual care suggested a similar magnitude of effect (SMD=–0.32, 95% CI –0.54 to –0.1; *I*^2^=63%; *P*<.001). SWiM was conducted for 4 trials. Of these, 2 were ICBT trials; 1 showed significant improvement in the percentage of participants with lower depression compared with usual care [24], and 1 study reported significant within-group improvement in the intervention group but no significant between-group differences after an active control (a website-based discussion forum) was used [33]. Trials of problem-solving therapy [39] and stress management [31] revealed no significant effect on depression compared to usual care.

#### Anxiety

Eleven studies measured participants’ anxiety level, using the State-Trait Anxiety Inventory Form Y [24], National Institutes of Health Patient Reported Outcomes Measurement Information System anxiety short form [36], Cardiac Anxiety Questionnaire [33-35,37-39], State Anxiety Inventory [40], HADS [42], and DASS [31].

**Figure 4:**
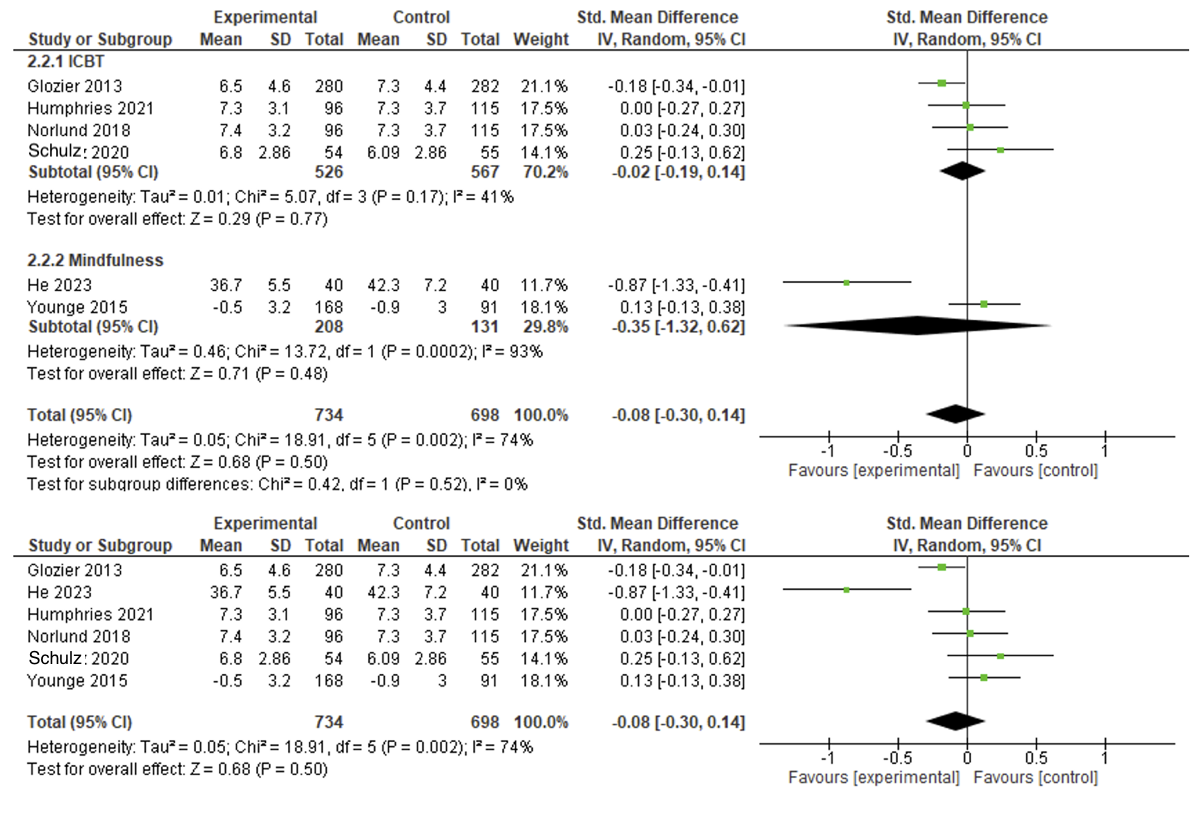
Forest plot showing the efficacy of eHealth psychological interventions for anxiety.

Data pooling of 6 of these trials showed no significant improvement in anxiety symptoms among participants receiving psychological eHealth interventions based on ICBT or mindfulness [42] (SMD=–0.08, 95% CI –0.13 to 0.14; *I*^2^=74%; *P*=.50; Figure 4). SWiM was conducted for 5 trials. Of these, 1 trial provided ICBT with other lifestyle promotion and showed a significant reduction in anxiety level when compared to usual care (*P*<.01) [24] and 2 others that also provided ICBT alone showed no significant improvement in anxiety level compared to usual care [36] or a website-based discussion forum [33]. Problem-solving therapy [39] and stress management [31] had no significant effect on anxiety level compared to usual care.

#### Stress

One trial delivering mindfulness training revealed no significant improvement in stress level as measured by the Perceived Stress Scale compared to usual care [42]. In another trial delivering stress management, no significant improvement in stress level measured by the DASS was observed compared to usual care [31].

#### Health-Related Quality of Life

**Figure 5:**
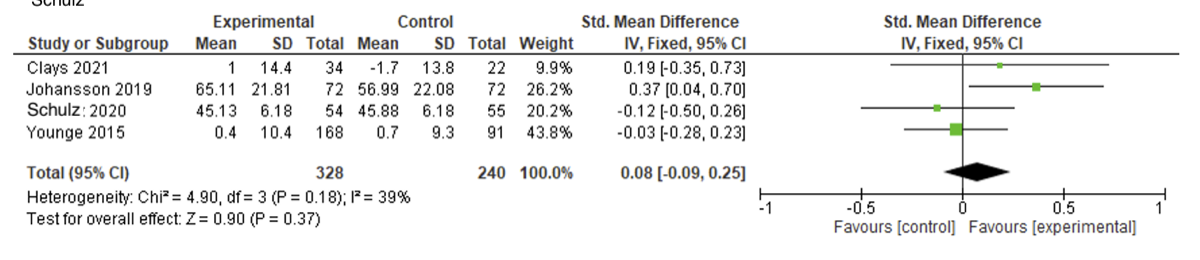
Forest plot showing the efficacy of eHealth-based psychological interventions for health-related quality of life.

Figure 5 displays a forest plot for the pooled results on health-related quality of life (HRQoL). Eight studies measured HRQoL, using either the Minnesota Living with Heart Failure Questionnaire [24,33], Short Form 12 [31,36,39], Short Form 36 [38,42], or Euro Qol Visual Analogue Scale [41]. Data pooling of 4 trials reporting an overall score or the mental dimension (where the overall score was unavailable) revealed no significant improvement in HRQoL (SMD=0.08, 95% CI –0.09 to 0.25; *I*^2^=39%; *P*=.37) compared to usual care [24,38,42] or a website-based discussion forum [41]. Trials testing ICBT [33,36], problem-solving therapy [39], and stress management [31] showed no significant effect on HRQoL when compared with usual care or a website-based discussion forum [33].

### Adverse Events and Cardiovascular Events

Three studies that delivered ICBT recorded participants at risk of deliberate self-harm, as detected by a validated depression scale, and reported similar numbers (n=21 in the intervention group and n=23 in the control group [37] vs n=2 in the intervention group and n=3 in the control group [35]) or fewer numbers (n=1 in the intervention group and n=2 in the control group) [41].

Four trials comparing ICBT to usual care reported cardiovascular outcomes. One reported significant reductions in 1-year and 3-year mortality risk within the group but not between groups [24]; another lower hospitalization-related costs, although this was nonsignificant (–$1010, 95% CI –$3294 to $1274; *P*=.39) [36]; yet another similar frequencies of hospitalization [38], and finally, another a nonsignificantly higher number of cardiovascular events (intervention group: n=36 cases; control group: n=25 cases) [34]. One study providing mindfulness training reported 5 and 10 participants with fluctuations of >50 mmHg in systolic blood pressure in the intervention and control groups, respectively [40].

## Discussion

### Overview

The aim of this review was to assess the effects of eHealth psychological interventions in improving psychological outcomes among individuals with CVDs. This is the first review of such interventions in CVD demonstrating they effectively reduce depression. The significant improvement identified could be attributed to the use of structured, evidence-based interventions, chiefly ICBT compared to usual care, website-based discussion forums, or education. These findings are consistent with other reviews in more general populations or those with other chronic conditions on the benefits of ICBT [43]. While the effects of ICBT on anxiety were not significant, more data on the effects of other eHealth psychological interventions, as well as their effects on stress, HRQoL, and cardiovascular events, are needed prior to drawing conclusions.

The effectiveness of eHealth psychological interventions in reducing depression among CVD patients is noteworthy. This finding is consistent with previous literature that reported the effectiveness of traditional (ie, non–technology-based) nonpharmacological psychological interventions in alleviating depression among CVD patients [44]. Another similarity is that CBT was identified as the most commonly used psychological therapy for CVD patients, followed by mindfulness and other stress management interventions [44,45]. However, previous CVD meta-analyses of traditional psychological intervention showed inconsistent clinical outcomes, including cardiac events and mortality [45,46]. One review found a small but nonsignificant effect of nonpharmacological psychological interventions in reducing cardiac mortality (risk ratio 0.80, 95% CI 0.64 to 1.00; *I*^2^=0%; *P*=.56) for patients with coronary heart disease [47]. Conversely, one meta-analysis suggested significant improvement in cardiac mortality (risk ratio 0.81, 95% CI 0.68 to 0.96) and the occurrence of cardiac events among participants who received psychological interventions compared to control group participants [45]. In this review, the clinical outcomes were not assessed or reported by most of the included studies, which hindered evaluation of the effect of eHealth psychological intervention for this parameter.

The impact of eHealth interventions on anxiety and HRQoL were inconclusive. This may be because the applied interventions did not fully address the inherent complexities of anxiety or the multifaceted nature of HRQoL faced by patients with CVDs [48]. For anxiety, specific therapeutic strategies—such as exposure therapy or specialized CBT approaches tailored and proven effective for anxiety management [49]—were not the primary focus of these trials. Additionally, improving HRQoL in CVD goes beyond psychological well-being to include improvement in physical endurance, social engagement, and daily functioning [48]. Effective interventions would need to integrate elements such as physical rehabilitation exercises, social skills training, and occupational therapy to holistically address HRQoL. This highlights a gap in the current therapeutic approaches and underscores the necessity for more integrative eHealth intervention designs tailored to such needs of CVD patients. The varied effectiveness likely also stems from the differing technology used and guidance levels in the interventions. For instance, mindfulness has been shown to be effective in face-to-face sessions [50], but its key strengths—deep interaction and personalized adjustment—may not translate as effectively to eHealth formats.

This review supports recommendation of ICBT as a standard treatment for depression in CVD patients [51]. However, considering a previous review focusing on face-to-face CBT that showed comprehensive and significant improvements in depression as well as anxiety and HRQoL among patients with CVDs [52], patient concerns or diagnoses and preferences must be considered. Two factors might explain the differences: (1) the prior review focused exclusively on participants diagnosed with mental disorders, whereas this review included a broader demographic of CVD patients; and (2) the inherent interpersonal interactivity and potential personalization of face-to-face CBT, which can help patients recognize and modify unhelpful thinking patterns, might be superior to online portals and discussion forums. Despite this, the resource-intensive and costly aspects of traditional CBT [53], coupled with the dynamic nature of mental health conditions among participants, highlight the potential of alternative delivery methods such as eHealth.

Moreover, a deeper examination of the eHealth modalities used by the studies included in this review reveals several unique strengths compared to traditional methods. First, the temporal attributes of some ICBT interventions, which allowed dynamic telemonitoring and daily tracking of heart rate, skin response, temperature, and physical activity levels, as well as support systems to provide tailored advice based on participants’ telemonitoring results and progress, are promising [24]. Second, in some trials, intervention adherence was tracked using built-in website counters of online course participation, with professionals providing individualized feedback on participants’ ICBT assignments for cognitive restructuring and behavioral activation [24,33-38,41]. Cognitive restructuring requires that participants continuously complete homework, identifying and modifying detrimental thought patterns and engaging in positive and meaningful activities. Thus, supporting participants in practicing ICBT in their daily lives, coupled with online support from professionals through time, could be one unique strength of this mode, leading to sustainable effects [54]. Lastly, the eHealth modality allows for a confidential platform in which participants can reveal negative thinking patterns and professionals can monitor progress and provide timely feedback or treatment plan adjustments. Thus, eHealth offers unique strengths in accessibility, scalability, and personalization [55].

When harnessing the capabilities of eHealth, it is important to note that technology serves as a tool and cannot replace the therapeutic encounter and rapport. Mental health professionals play a crucial role as gatekeepers in the creation and implementation of eHealth psychological interventions [5,56]. Their analysis of patients’ self-monitoring data and provision of real-time feedback are crucial for quality care and outcome optimization. Additionally, these professionals are instrumental in tailoring treatment to the patient’s context, such as by managing workplace stress or other inciting factors in their physical and psychosocial environments, thereby enhancing intervention applicability and impact [56].

### Implications

Some practice implications are important to consider. First, many CVD patients continue to have unrecognized comorbid psychological conditions. With more patients adopting technology and with technological advances, the adoption, acceptance, quality, and efficacy of psychological eHealth interventions may improve. Second, it may be important to be more selective in allocating patients to psychological eHealth interventions, such that patients who do not prefer technology, or who have no (or who have severe) psychological conditions should be provided with alternative interventions. Finally, in addition to considering patient preference, stepped care, such as was implemented in one included trial, should be pursued so nonresponding patients can receive care through to remission and so that their excess cardiovascular risk is reduced [36].

Several implications for future studies are noteworthy. Studies may consider combining ICBT with antidepressants, as guidelines from the American Heart Association and American Academy of Family Physicians recommend such combination for the treatment of post–acute coronary syndrome depression [57,58], addressing both psychological and physiological causes as well as outcomes of depression in this population. Strategies such as enhancing eHealth platform interactivity for real-time feedback, using adaptive algorithms for personalized therapy, and integrating virtual or augmented reality for immersive experiences could enable achievement of comparable benefits to traditional therapeutic sessions [59].

### Limitations

The review is limited by the limited available trials in this field. Moreover, while publication bias was evaluated by comparing findings with registered trials and published protocols, meta-regression and funnel plot asymmetry assessment for publication bias were not possible. The participants were predominantly middle-aged or young-older adults from developed countries, which may limit the generalizability of the findings to older age groups or cultures unfamiliar with Western psychological treatment approaches. Caution is needed in interpreting the results and replicating the interventions, as most included studies used mixed eHealth approaches, combining multiple intervention modalities. Future studies should also ensure best practices are applied in terms of randomization, allocation, and blinding methods to enhance evidence quality.

### Conclusion

This first review of eHealth-based psychological interventions for CVD patients found that these interventions, particularly ICBT, significantly reduced depressive symptoms but not anxiety, with unclear effects on stress, HRQoL, and cardiovascular events. Research is urgently needed to assess the long-term impact, scalability, and accessibility of these interventions, which could play a transformative role in improving psychological well-being in this population. Collaboration among researchers, policy makers, and multidisciplinary cardiovascular health professionals is critical to advancing this field. Incorporating persuasive design elements, such as goal setting and motivational feedback, harnessing the temporal (ie, time-sensitive) intervention attributes of eHealth, and involving mental health professionals will be key to maximizing the effectiveness of eHealth approaches for CVD care.

## Appendix

Multimedia Appendix 1 Search strategies, characteristics of included trials (n=12), and recruitment and attrition rate of included studies.

Multimedia Appendix 2 PRISMA checklist.

## Abbreviations

CONSORT: Consolidated Standards of Reporting Trials
CVD: cardiovascular disease
DASS: Depression, Anxiety, and Stress Scale
HADS: Hospital Anxiety and Depression Scale
HRQoL: health-related quality of life
ICBT: internet-based cognitive behavioral therapy
MeSH: Medical Subject Headings
PICOS: populations, interventions, comparisons, outcomes, and study designs
PRISMA: Preferred Reporting Items for Systematic Reviews and Meta-Analyses
SMD: standardized mean difference
SWiM: synthesis without meta-analysis

## Data Availability

Data sharing is not applicable to this systematic review and meta-analysis as no new data were created in this study.
