# Supplementary material for: Psychological eHealth Interventions for Patients With Cardiovascular Diseases: Systematic Review and Meta-Analysis
Source: J Med Internet Res. 2025 Apr 7;27:e57368. doi: 10.2196/57368 (PMC12012401; doi:10.2196/57368)
Supplement: Multimedia Appendix 1 [file jmir_v27i1e57368_app1.docx]

**Supplementary Material 1.**

Search strategies for Embase

((('cardiovascular disease*' OR 'angina' OR 'heart infarction'/exp OR 'heart infarction' OR 'peripheral vascular disease'/exp OR 'peripheral vascular disease' OR 'heart disease'/exp OR 'heart disease' OR 'heart failure'/exp OR 'heart failure' OR 'coronary artery disease'/exp OR 'coronary artery disease' OR 'heart muscle ischemia'/exp OR 'heart muscle ischemia' OR 'coronary thrombo*' OR 'acute coronary syndrome*' OR 'coronary bypass'/exp OR 'coronary bypass' OR cabg OR 'percutaneous coronary intervention'/exp OR 'percutaneous coronary intervention' OR pci OR 'angina pectoris'/exp OR 'angina pectoris' OR 'angina'/exp OR angina OR ischeam* OR 'heart disorder*') AND ('mind-body' OR 'relaxation'/exp OR relaxation OR counsel* OR 'cognitive therapy'/exp OR 'cognitive therapy' OR 'behavior therapy'/exp OR 'behavior therapy' OR 'meditation'/exp OR 'meditation' OR 'positive psychology'/exp OR 'positive psychology' OR 'psychotherapy'/exp OR 'psychotherapy' OR 'relaxation training'/exp OR 'relaxation training') AND ('mobile phone'/exp OR 'mobile phone' OR 'cell phone use'/exp OR 'cell phone use' OR 'smartphone'/exp OR smartphone OR 'text messaging'/exp OR 'text messaging' OR 'chatbot'/exp OR chatbot OR 'web'/exp OR web OR 'internet'/exp OR internet OR 'telehealth'/exp OR telehealth OR 'video consultation'/exp OR 'video consultation')) AND ('feasibility study'/de OR 'pilot study'/de OR 'randomized controlled trial'/de OR 'randomized controlled trial topic'/de)) AND ('article'/it OR 'article in press'/it)

Search strategies for PubMed

| **Query** | **Limiters/Expanders** | **No. of results** |
| --- | --- | --- |
| S4 | S1 AND S2 AND S3 | 734 |
| S3 | (MH "Randomized Controlled Trials+") OR (MH "Intervention Trials") | 288,080 |
| S2 | (MH "Cellular Phone+") OR (MH "Behavior Therapy+") OR (MH "Relaxation") OR (MH "Relaxation Techniques+") OR (MH "Muscle Relaxation") OR (MH "Positive Psychology") OR (MH "Psychotherapy, Group+") OR "'mind-body' OR relaxation OR counsel* OR 'cognitive therapy' OR 'behavior therapy' OR meditation OR relaxation OR 'positive psychology' OR psychotherapy OR 'mobile phone' OR smartphone OR text" | 101,659 |
| S1 | (MH "Heart Diseases+") OR (MH "Coronary Disease+") OR (MH "Myocardial Ischemia+") OR (MH "Myocardial Infarction+") OR (MH "Peripheral Vascular Diseases+") OR (MH "Coronary Arteriosclerosis") OR (MH "Cardiovascular Diseases+") OR ""cardiovascular disease* OR angina OR 'heart infarction' OR 'peripheral vascular disease*' OR 'heart disease' OR 'coronary artery disease' OR heart muscle ischemia' OR 'acute coronary syndrome' OR 'coronary bypass' OR 'heart disorder'" | 772,629 |

Search strategy for Medline

| **Query** | **Search terms** | **No of results** |
| --- | --- | --- |
| 1 | "cardiovascular disease* ".m_titl. | 44,506 |
| 2 | angina.m_titl. | 18,629 |
| 3 | 'heart infarction'.m_titl. | 95 |
| 4 | "'peripheral vascular disease*'".m_titl. | 2,381 |
| 5 | 'heart disease*'.m_titl. | 66,382 |
| 6 | "heart failure".m_titl. | 83,001 |
| 7 | 'coronary artery disease'.m_titl. | 31,142 |
| 8 | 'heart muscle ischemia'.m_titl. | 1 |
| 9 | "'coronary thrombo*'".m_titl. | 1,246 |
| 10 | "'acute coronary syndrome*'".m_titl. | 17,302 |
| 11 | 'coronary bypass'.m_titl. | 3,400 |
| 12 | 'percutaneous coronary intervention'.m_titl. | 14,639 |
| 13 | 'angina pectoris'.m_titl. | 8,613 |
| 14 | "'heart disorder*'".m_titl. | 99 |
| 15 | 1 or 2 or 3 or 4 or 5 or 6 or 7 or 8 or 9 or 10 or 11 or 12 or 13 or 14 | 276,768 |
| 16 | 'mind-body'.m_titl. | 1,049 |
| 17 | relaxation.m_titl. | 19,086 |
| 18 | "counsel*".m_titl. | 21,867 |
| 19 | 'cognitive therapy'.m_titl. | 1,322 |
| 20 | 'behavior therapy'.m_titl. | 2,225 |
| 21 | meditation.m_titl. | 2,364 |
| 22 | 'positive psychology'.m_titl. | 320 |
| 23 | psychotherapy.m_titl. | 17,846 |
| 24 | 'mobile phone'.m_titl. | 2,490 |
| 25 | 'cell phone use'.m_titl. | 134 |
| 26 | smartphone.m_titl. | 6,998 |
| 27 | 'text messaging'.m_titl. | 801 |
| 28 | chatbot.m_titl. | 365 |
| 29 | internet.m_titl. | 17,525 |
| 30 | telehealth.m_titl. | 5,485 |
| 31 | 'video consultation'.m_titl. | 75 |
| 32 | 16 or 17 or 18 or 19 or 20 or 21 or 22 or 23 or 24 or 25 or 26 or 27 or 28 or 29 or 30 or 31 | 98,852 |
| 33 | 'randomized controlled trial'.m_titl. | 58,484 |
| 34 | 15 and 32 and 35 | 74 |

Search strategy for Cochrane library

| #1 | relax* OR 'mind-body' OR Relaxation OR counsel* OR 'cognitive therapy' OR 'behavior therapy' OR 'meditation' OR 'positive psychology' OR 'psychotherapy' | 146786 |
| --- | --- | --- |
| #2 | 'mobile phone' OR 'mobile phone' OR 'cellphone' OR 'smartphone' OR smartphone OR 'text messaging' OR chatbot OR web OR 'internet' OR 'telehealth' OR 'video consultation' | 44238 |
| #3 | 'cardiovascular disease*' OR 'angina' OR 'heart infarction' OR 'peripheral vascular disease' OR 'heart disease' OR 'heart failure' OR 'coronary artery disease' OR 'heart muscle ischemia' OR 'coronary thrombo*' OR 'acute coronary syndrome*' OR 'coronary bypass' OR cabg OR 'percutaneous coronary intervention' OR pci OR 'angina pectoris' OR 'angina pectoris' OR 'angina' OR ischeam* OR 'heart disorder*' | 175913 |
| #4 | #1 AND #2 AND #3 | 1864 |

Search strategy for CINAHL

| **#** | **Query** | **Results** |
| --- | --- | --- |
| S5 | ((MH "Cognitive Therapy+") OR (MH "Behavior Therapy+") OR (MH "Positive Psychology") OR (MH "Manipulation, Psychological") OR (MH "Cognitive Restructuring") OR (MH "Cognitive Therapy (Iowa NIC)+") OR (MH "Reinforcement (Psychology)+") OR (MH "Dialectical Behavior Therapy") OR (MH "Adaptation, Psychological+") OR (MH "Behavior Therapy (Iowa NIC)+") OR (MH "Muscle Relaxants, Central+") OR (MH "Models, Psychological+") OR (MH "Psychological Theory+") OR "psychotherap* OR psycholog* OR relax* OR “Mind-Body OR Relaxation OR counsel* OR “Cognitive Therapy” OR Behavior Therapy OR Meditation OR meditat* OR “positive psychology”" OR (MH "Relaxation Techniques+") OR (MH "Mind Body Techniques+") OR (MH "Muscle Relaxation") OR (MH "Stress, Psychological+") OR (MH "Recreational Therapy") OR (MH "Music Therapy (Iowa NIC)") OR (MH "Music Therapy") OR (MH "Dance Therapy") OR (MH "Art Therapy (Iowa NIC)") OR (MH "Art Therapy") OR (MH "Relaxation") OR (MH "Progressive Muscle Relaxation (Iowa NIC)") OR (MH "Psychological Techniques+") OR (MH "Psychology+") OR (MH "Psychological Distress") OR (MH "Acceptance and Commitment Therapy") OR (MH "Mentalization-Based Therapy") OR (MH "Home Physical Therapy") OR (MH "Psychophysiology+") OR (MH "Rehabilitation, Psychosocial+") OR (MH "Psychosocial Aspects of Illness+") OR (MH "Psychosocial Intervention")) AND (S1 AND S2 AND S3) | 3,373 |
| S4 | ((MH "Cognitive Therapy+") OR (MH "Behavior Therapy+") OR (MH "Positive Psychology") OR (MH "Manipulation, Psychological") OR (MH "Cognitive Restructuring") OR (MH "Cognitive Therapy (Iowa NIC)+") OR (MH "Reinforcement (Psychology)+") OR (MH "Dialectical Behavior Therapy") OR (MH "Adaptation, Psychological+") OR (MH "Behavior Therapy (Iowa NIC)+") OR (MH "Muscle Relaxants, Central+") OR (MH "Models, Psychological+") OR (MH "Psychological Theory+") OR "psychotherap* OR psycholog* OR relax* OR “Mind-Body OR Relaxation OR counsel* OR “Cognitive Therapy” OR Behavior Therapy OR Meditation OR meditat* OR “positive psychology”" OR (MH "Relaxation Techniques+") OR (MH "Mind Body Techniques+") OR (MH "Muscle Relaxation") OR (MH "Stress, Psychological+") OR (MH "Recreational Therapy") OR (MH "Music Therapy (Iowa NIC)") OR (MH "Music Therapy") OR (MH "Dance Therapy") OR (MH "Art Therapy (Iowa NIC)") OR (MH "Art Therapy") OR (MH "Relaxation") OR (MH "Progressive Muscle Relaxation (Iowa NIC)") OR (MH "Psychological Techniques+") OR (MH "Psychology+") OR (MH "Psychological Distress") OR (MH "Acceptance and Commitment Therapy") OR (MH "Mentalization-Based Therapy") OR (MH "Home Physical Therapy") OR (MH "Psychophysiology+") OR (MH "Rehabilitation, Psychosocial+") OR (MH "Psychosocial Aspects of Illness+") OR (MH "Psychosocial Intervention")) AND (S1 AND S2 AND S3) | 8,466 |
| S3 | (MH "Cognitive Therapy+") OR (MH "Behavior Therapy+") OR (MH "Positive Psychology") OR (MH "Manipulation, Psychological") OR (MH "Cognitive Restructuring") OR (MH "Cognitive Therapy (Iowa NIC)+") OR (MH "Reinforcement (Psychology)+") OR (MH "Dialectical Behavior Therapy") OR (MH "Adaptation, Psychological+") OR (MH "Behavior Therapy (Iowa NIC)+") OR (MH "Muscle Relaxants, Central+") OR (MH "Models, Psychological+") OR (MH "Psychological Theory+") OR "psychotherap* OR psycholog* OR relax* OR “Mind-Body OR Relaxation OR counsel* OR “Cognitive Therapy” OR Behavior Therapy OR Meditation OR meditat* OR “positive psychology”" OR (MH "Relaxation Techniques+") OR (MH "Mind Body Techniques+") OR (MH "Muscle Relaxation") OR (MH "Stress, Psychological+") OR (MH "Recreational Therapy") OR (MH "Music Therapy (Iowa NIC)") OR (MH "Music Therapy") OR (MH "Dance Therapy") OR (MH "Art Therapy (Iowa NIC)") OR (MH "Art Therapy") OR (MH "Relaxation") OR (MH "Progressive Muscle Relaxation (Iowa NIC)") OR (MH "Psychological Techniques+") OR (MH "Psychology+") OR (MH "Psychological Distress") OR (MH "Acceptance and Commitment Therapy") OR (MH "Mentalization-Based Therapy") OR (MH "Home Physical Therapy") OR (MH "Psychophysiology+") OR (MH "Rehabilitation, Psychosocial+") OR (MH "Psychosocial Aspects of Illness+") OR (MH "Psychosocial Intervention") | 291,186 |
| S2 | (MM "Myocardial Ischemia") OR (MM "Coronary Disease") OR (MH "Coronary Arteriosclerosis") OR (MH "Heart Diseases+") OR (MH "Pulmonary Heart Disease") OR (MH "Syndrome X") OR (MH "Myocardial Infarction+") OR (MH "Coronary Artery Bypass+") OR (MH "Cardiac Patients") OR (MH "Angioplasty, Transluminal, Percutaneous Coronary") OR "'cardiovascular disease*' OR 'angina' OR 'heart infarction' OR 'peripheral vascular disease' OR 'heart disease' OR 'heart failure' OR 'coronary artery disease' OR 'heart muscle ischemia' OR 'coronary thrombo*' OR 'acute coronary syndrome*' OR 'coronary bypass' OR cabg OR 'percutaneous coronary intervention' OR pci OR 'angina pectoris' OR 'angina pectoris' OR 'angina' OR ischeam* OR 'heart disorder*'" OR (MH "Heart Defects, Congenital+") OR (MH "Acute Coronary Syndrome") OR (MH "Peripheral Vascular Diseases+") OR (MH "Coronary Aneurysm") OR (MH "Coronary Stenosis+") OR (MH "Coronary Vasospasm+") OR (MH "Intracranial Arterial Diseases+") OR (MH "Heart Failure+") OR (MH "Percutaneous Coronary Intervention") OR (MH "Cerebrovascular Disorders+") OR (MH "Coronary Artery Calcification") OR (MH "Heart Catheterization+") OR (MH "Angina, Stable") OR (MH "Angina Pectoris+") OR (MH "Coronary Thrombosis") OR (MH "Coronary Restenosis") OR (MH "Cerebral Arterial Diseases+") OR (MH "Heart Neoplasms+") OR (MH "Coronary Vessel Anomalies+") OR (MH "Cardiovascular Diseases+") OR (MH "Vascular Diseases+") OR (MH "Heart+") OR (MH "Coronary Occlusion+") OR (MH "Coronary Circulation") OR (MH "Coronary Care Units") OR (MH "Coronary Angiography") OR (MH "Cardiac Valve Annuloplasty+") OR (MH "Heart Valves+") OR (MH "Heart Sounds") OR (MH "Heart, Artificial") OR (MH "Heart Septal Defects+") OR (MH "Heart Rate+") OR (MH "Heart Murmurs") OR (MH "Heart Function Tests+") OR (MH "Heart Block+") OR (MH "Angina, Unstable") OR (MH "Psychosocial Aspects of Illness+") OR (MH "Peripheral Nerves+") | 969,889 |
| S1 | (MM "Videorecording") OR (MM "World Wide Web Applications") OR (MM "Smartphone") OR (MM "Telehealth+") OR (MM "Telephone+") OR (MM "Text Messaging+") OR "'mobile phone' OR 'mobile phone' OR 'cellphone' OR 'smartphone' OR smartphone OR 'text messaging' OR chatbot OR OR web OR 'internet' OR 'telehealth' OR 'video consultation'" OR (MH "Mobile Applications") OR (MH "Internet-Based Intervention") OR (MH "Telecommunications+") OR (MH "Remote Consultation") OR (MH "Referral and Consultation+") OR (MH "Instant Messaging") OR (MH "Internet Connections") OR (MH "Web Browsers") OR (MH "Peer Counseling") OR (MH "Counseling+") OR (MH "Teaching, Guidance, and Counseling (Omaha)") OR (MH "Internet Access") OR (MH "Webcasts+") OR (MH "Counseling (Iowa NIC)") OR (MH "Internet+") OR (MH "Audiovisuals+") OR (MH "Videoconferencing+") | 431,777 |

**Supplementary material 2. Characteristics of included trials (n=12)**

| no. | Author (Year) | Country | Gender (female/ total sample) | N (IG/CG) | Mean age | Setting | Population | Intervention | Interveners | Control condition | Intervention adherence | Data collection timepoint |
| --- | --- | --- | --- | --- | --- | --- | --- | --- | --- | --- | --- | --- |
| Internet-based Cognitive-Behavior Therapy | | | | | | | | | | | | |
| 1 | Clays (2021) | Belgium | 0.232 | 61 (38/23) | 63 | Three hospitals in Belgium | Congestive heart failure | ICBT Personal Health System  Duration: 3 to 6 months, individualized. **Modules:** Physical health management (exercise, nutrition, self-monitoring, disease education); Psychological health (CBT, mindfulness, psychological counselling), all delivered by mobile application connected to sensing devices.. **Support/Technology**: Sensing devices included wristband to record hear rate, skin response, temperature and physical activity level, with additional monitor for blood pressure and weight; Decision Support system (DSS) to analyze the integrated data to provide personalized health management advice, and adjust advice based on patients progress and feedback. | Initiated during a home visit by research team members | Usual care with standardized care in line with clinical guidelines offered by cardiologist, general practitioners and CHF nurse | Not reported, but technical issues had affected their adherence | Baseline, post-intervention |
| 2 | Davidson (2013) | USA | 0.353 | 150 (73/77) | 59.6 | 2 private and 5 academic ambulatory centers across the United States. | Acute coronary syndrome with elevated depression after | ICBT Problem-Solving Sessions  Duration: 24 weeks (approximately 6 months)  Modules: problem solving treatment offered via telephone or internet, PST is a manualized CBT on teaching how to solve personal problems leading to depressive symptoms  Support/technology: telephone and internet delivery to provide remote intervention with a team of healthcare professionals to monitor progress; with adjustment to plan every 6-8 weeks based on patient progress. | A combination of healthcare professionals including study physicians, nurse practitioners, and local health care providers. | Usual care informed by physician about their participation in the trial and was free to obtain any depression care from any health care provider | Not reported | Baseline, post-intervention |
| 3 | Glozier (2013) | Australia | 0.618 | 562 (280/282) | 57.95 | Recruited from research study | A mixture of patients with CVD and those at high CVD risks, with mild to moderate depression | ICBT 'E-Couch' Program  Duration: 2 weeks  Module: ICBT with core component involving cognitive restructuring and behavioral activation; interpersonal psychotherapy (IPT) with techniques aimed at improving interpersonal relationships which can affect mood and depression; psychoeducation to provide mental health literacy which is crucial for managing symptoms of depression.  Support/technology: website platform “E-coach” that users could access therapy module online, with tracking of progress to enhance adherence and engagement | Self-guided therapy, participants worked at their own pace | Active control - Healthwatch 12-week online education program on nutrition, stroke, physical activities, medicines at home, blood pressure, cholesterol without therapeutic content. | 73% completed the intervention | Baseline, 4-week post-baseline, 8-week post-baseline, post-intervention |
| 4 | Humphries (2021) | Sweden | 0.335 | 239 (117/122) | 59.6 | Cardiac clinics | Post-acute myocardial infarction with mild to moderate depression | ICBT Therapist-Guided Program  Duration: 14 weeks. Modules: 11, including worry and fear management, behavioral activation, problem-solving, communication, relaxation, insomnia, life values, and relapse prevention. Each patient had access to a therapist who guided them through modules, provide feedback and support as needed, they were given tasks to complete outside the therapy sessions.  Support/technology: therapy was delivered through secure web portal (U-Care) which allow patients interacted with therapist, supported with various media such as video and text available in library. Portal would track patient engagement and progress and therefore allow therapist to tailor intervention. | Therapist | Usual care included standard protocol secondary prevention and cardiac rehabilitation offered by the regional healthcare system | Just over half of the participants in the iCBT group completed the first introductory module, and only 15 continued to work through any of the remaining 10 modules due to lack of time, technical aspects (such as insufficient computer literacy), and unpleasant emotions evoked by the intervention. | Baseline, 14-week post-baseline, 12 months after myocardial infarction |
| 5 | Johansson (2019) | Sweden | 0.381 | 144 (72/72) | 62.9 | Medical and cardiology clinics | Diagnosis of atrial fibrillation or atrial flutter, coronary heart disease, or heart failure with at least mild depression | ICBT Behavioral Program  Duration: 9 weeks. Modules: Goal setting, psychoeducation, problem-solving, behavioral activation. Support/technology: delivered thorugh an online platform, where patients accessed the program through this platform, with modules including text, short videos, and were supplemented by weekly assignment. Through this platform, nurses provided written feedback on weekly assignment, offered encouragement and addressed any questions through messaging function, with questions answered within 24 hours on working days. Regular screenings for suicidality were conducted to ensure patient safety. | Nurses with experience in cardiovascular disease | Active control - 9-week web-based moderated discussion forum, with new discussion topic each week | 60% completed all 7 modules, 82% completed > 50% of modules | Baseline, post-intervention |
| 6 | Lundgren (follow-up Westas) (2016) | Sweden | 0.61 | 50 (25/25) | 62.9 | Outpatient | Heart failure with at least mild depressive symptoms | ICBT Guided Program for Heart Failure:  Duration: 9 weeks. Modules:7 modules covering topics like heart failure management, depressive symptoms, relaxation exercises, behavior activation, problem-solving, and a summary module to reinforce learned content. Support/technology: modules delivered through secure web portal. Participants received written feedback on each assignment from nurse, and tracked participants logins and module completion, providing data on engagement and adherence. | Mental health specialist nurse with experience in heart failure care. | Active control - Web-based moderated discussion forum, participants made their posts in discussion thread on each topic | not reported | Baseline, post-intervention |
| 7 | Norlund (2018) | Sweden | 0.335 | 239 (117/122) | 60 | 4 hospitals in Sweden | Recent myocardial infarction with depression or anxiety symptoms | ICBT Therapist-Guided Psychoeducation Program  Duration: 14 weeks. Modules: Managing worry, behavioral activation, relaxation techniques, coping with insomnia, relapse prevention, and personal values. Support/technology: Online U-Care portal to deliver the iCBT content, licensed psychologists provided guidance and feedback on homework through portal, modules included PDFs with text-based psychoeducation and multimedia elements, library with videos and readings to support module themes, and also discussion board allowed interaction with other participants. | Licensed psychologists in consultation with patients who had a history of depression and anxiety post-myocardial infarction (MI). | Treatment as usual | 38.4% completed introductory module only, and 15.4%  completed additional modules, only one participant completed a 14-week treatment | Baseline, post-intervention |
| 8 | Schulz (2020) | Germany | 0.186 | 118 (59/59) | 58.8 | Seven medical centers/ hospitals | Patients with implantable cardioverter-defibrillator with increased anxiety and depression | ICBT Web-Based Group Intervention Duration: 6 weeks. Modules: education information on medical, technical and psychosical issues related to living with ICD, based on CBT principles to include tools for challenging irrational thoughts, planning positive activities and resource-oriented problem-solving; a virtual self-help group where participants could share experiences and support each other  Support/ technology: intervention delivered through web portal “ICD-Forum.de” with interactive element like two-column technique for modifying thoughts, with other CBT-based tools, on-demand support from trained clincial psychologist through open discussion board and peer communication; custom-designed interface displaying weekly topics, psychologist contact and patient discussion board and emergency plans. | Trained clinical psychologist | Usual care | Not reported | Baseline, immediate after intervention (6-week), 1-year follow-up |
| Problem-solving strategy | | | | | | | | | | | | |
| 9 | Habibović (2017) | Netherlands | 0.187 | 289 (146/143) | 58.5 | Implanting center at hospital | Patients with implantable cardioverter defibrillators | Problem-Solving Therapy Duration: Self-paced, 12-week web-based program. Modules: 6 sessions of psychoeducation related to ICDs. Support/Technology: delivered through online platform “WEBCARE” including structured lessons and patients received personalized feedback from their coaches via the websites. Participants provided with a CD with relaxation exercise to support stress management throughout study. Automated biweekly reminder was sent via emails for homework submission. | Trained master-level psychologists, served as "coach" | Usual care | 23.3% completed full intervention, 16.5% never logged on to the intervention | Baseline, 3-, 6-, 12 months post implantation |
| Mindfulness intervention | | | | | | | | | | | | |
| 10 | He (2023) | China | 0.2 | 84 (42/40) | 59.05 | First Affiliated Hospital of Nanjing Medical University, Nanjing, China | Patient with atrial fibrillation scheduled for Radiofrequency catheter ablation | Mindfulness meditation Intervention (App-Based) Duration: Single 35-minute session. Modules: guided mindfulness practices designed specifically for AF patients to help them relax during RFCA procedures without affecting the procedure itself, including breathing evenly, relaxing muscles and visualization.  Support/Technology: delivered via BCI-based mindfulness meditation App “Focus Zen”, including a headband that collect EEG data to monitor and respond to patients’ mental state during meditation, provides real-time feedback on patients’ brain state and adjust the app’s interface and headband light color accordingly; personalized audio guidance deliver meditation instruction and background sounds tailored to patients’ preferences; AI algorithm to analyze EEG data to classify and prompt patients current brain state. | Registered nurse | Routine care for ablation procedure and was informed about the procedure of ablation and characteristics of impending pain in ablation | Not reported | 30-min before ablation and 30-min after ablation |
| 11 | Younge (2015) | UK | 0.463 | 324 (215/109) | 43.2 | Outpatient cardiology clinic | Patients with heart disease (ischemic, valvular, congenital heart disease, or cardiomyopathy | Mindfulness Intervention (Web-Based) Duration: 12-week training. Modules: standardized online program including meditation, self-reflection and yoga. The program was self-directed to encourage independent practice. Practical assignment to incorporate mindfulness into daily life.  Support/Technology: intervention delivered through online delivery platform that they received reminders via emails and standardized text to encourage engagement, all patients were monitored through completion of program questions. | Self-directed | Usual care with regular outpatient visits, lifestyle advice on nutrition, smoking, exercise, stress reduction, medication | Not reported | Baseline |
| Stress management program | | | | | | | | | | | | |
| 12 | Lambert (pilot) (2022) | Canada | 0.559 | 59 (30/29) | Not reported | Three cardiovascular clinics | Physician-confirmed diagnosis of CVD, with moderate stress and at least 18 yo | Self-Directed Web-Based Stress Management Program  Duration: Two stages, duration not reported. Modules: stress dairy to monitor stress and triggers, physical activity tracking, deep breathing and relaxation techniques. CBT technique to manage and reframe negative thoughts, guides on improving sleep patterns and habits.  Support/Technology: delivered by web portal “My Health CheckUp” for accessing stress management modules, with slideshow presentations and practice-based components for each module, tracking progress and completion of modules, weekly telephone calls by couch to discuss progress, set goals and encourage engagement, additional support with motivational interviewing to enhance motivation, particularly for those with low response rate to web-based program. | Trained lay coach | Usual care with community-based services | Completed 1.7 out of 5 modules, favoring the stress diary; received an average of 4.4 coach calls, often extended beyond the intended duration due to technical difficulties; only 33% of participants received the minimum intended dose, with some declining or missing the calls | Baseline, 6-week after, 12-week baseline |

Note. IG = intervention group; CG = control group; ICBT, Internet-Based Cognitive Behavioral Therapy

**Supplementary material 3. Recruitment and attrition rate of included studies**

|  | Recruitment rate (%) | | | Attrition rate (%) | | | | | | Reasons for dropout | | | |
| --- | --- | --- | --- | --- | --- | --- | --- | --- | --- | --- | --- | --- | --- |
|  | Eligible  (n) | Randomized (n) | Recruitment (%) | Allocated to intervention group | Lost to  follow-up | Attrition rate in intervention group (%) | Allocated to control group | Lost to follow-up | Attrition rate in control group (%) | Health-Related (IG) | Non-Health-Related (IG) | Health-Related (CG) | Non-Health-Related (CG) |
| Lambert 2022 | 73 | 59 | 80.82 | 29 | 6 | 20.69 | 30 | 13 | 43.33 | NR |  |  |  |
| Lundgren 2016 | 80 | 50 | 62.50 | 25 | 4 | 16.00 | 25 | 5 | 20.00 |  | all |  | all |
| Clays 2021 | 79 | 65 | 82.28 | 38 | 4 | 10.53 | 23 | 1 | 4.35 | 1 | all |  | all |
| Humphries 2021 | 1982 | 239 | 12.06 | 117 | 28 | 23.93 | 122 | 10 | 8.20 | NR |  |  |  |
| Norlund 2018 | 1982 | 239 | 12.06 | 117 | 21 | 17.95 | 122 | 7 | 5.74 | NR |  |  |  |
| Davidson 2013 | 177 | 150 | 84.75 | 73 | 6 | 8.22 | 77 | 6 | 7.79 | 2 |  | 2 |  |
| Habibovic 2017 | 1024 | 289 | 28.22 | 146 | 35 | 23.97 | 143 | 19 | 13.29 | NR |  |  |  |
| Glozier 2013 | 1862 | 562 | 30.18 | 280 | 57 | 20.36 | 282 | 9 | 3.19 |  | all |  |  |
| Schulz 2020 | 1204 | 118 | 9.80 | 59 | 14 | 23.73 | 59 | 8 | 13.56 | 1 |  |  |  |
| He 2023 | 114 | 84 | 73.68 | 42 | 2 | 4.76 | 42 | 2 | 4.76 | 2 |  | 1 |  |
| Johanson 2019 | 272 | 144 | 52.94 | 72 | 7 | 9.72 | 72 | 10 | 13.89 |  | all |  |  |
| Younge 2015 | 1044 | 324 | 31.03 | 215 | 47 | 21.86 | 109 | 18 | 16.51 |  | all |  |  |
| ^a^ Defined as the percentage of individuals who were eligible and consented to participate  ^b^ Defined as the percentage of participants who consented but dropped out of the study  NR, Not reported; IG, intervention group; CG, control group | | | | | | | | | | | | | |
